# Supplementary material for: Metabolic maintenance of breast cancer cells and metastases through E-cadherin/YAP–dependent pyruvate carboxylase expression
Source: bioRxiv. 2026 Apr 16:2026.04.13.718309. Preprint. [Version 1] doi: 10.64898/2026.04.13.718309 (PMC13105095; doi:10.64898/2026.04.13.718309)
Supplement: Supplementary file 1 [file media-1.pdf]

Supplementary Figure S1

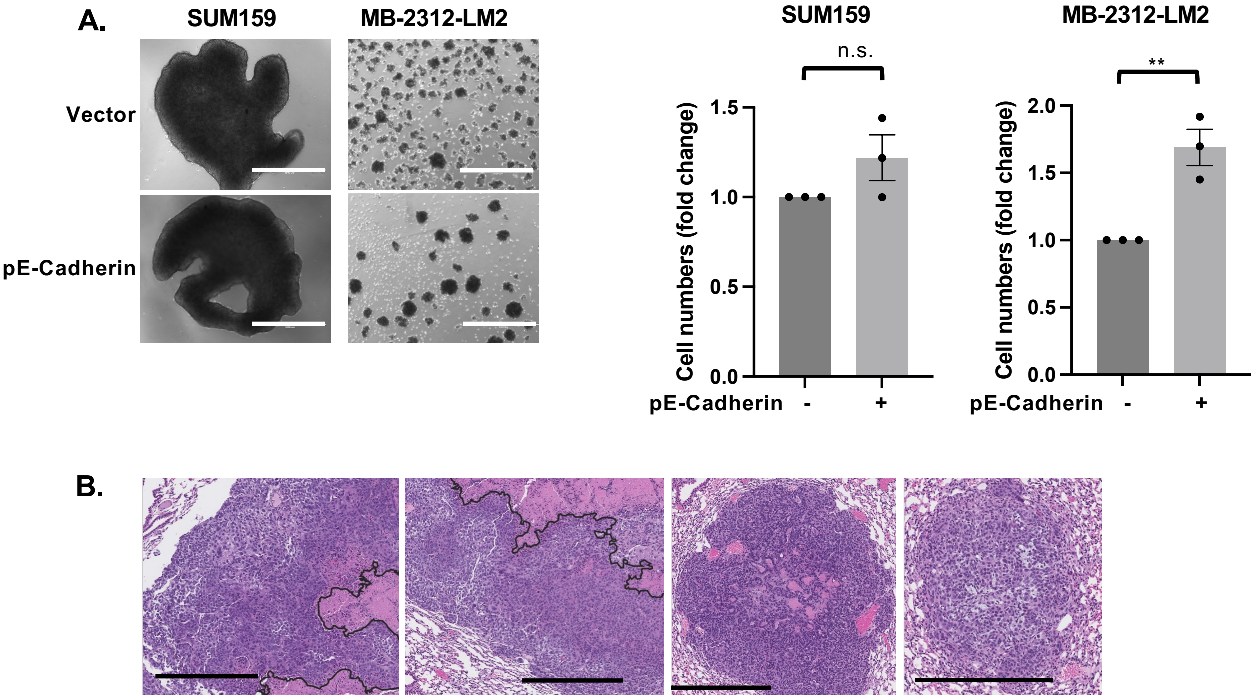

Figure S2

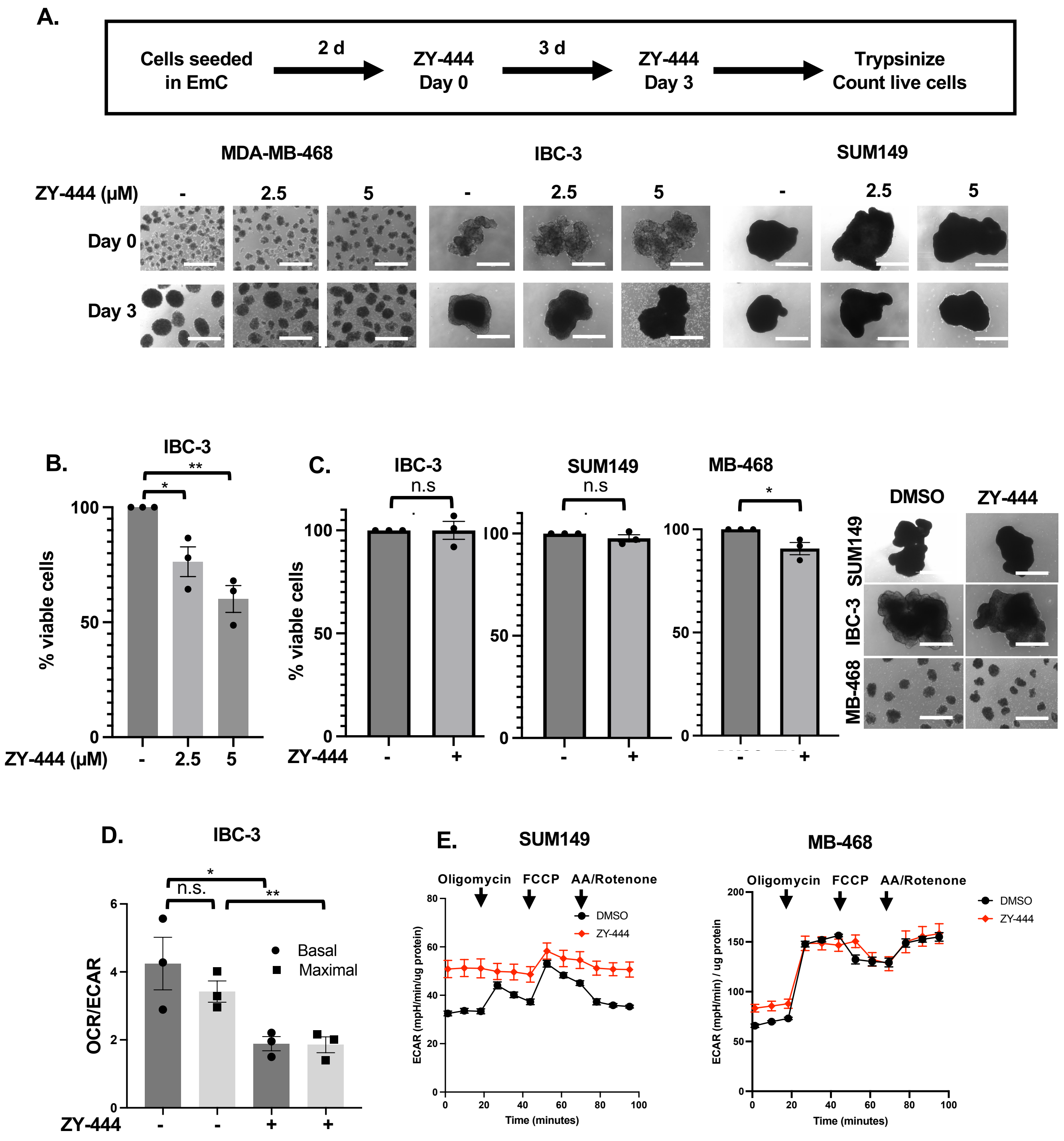
A.
Cells seeded in EmC
2 d
ZY-444 Day 0
3 d
ZY-444 Day 3
Trypsinize Count live cells
MDA-MB-468
IBC-3
SUM149
ZY-444 (µM)
-
2.5
5
Day 0
Day 3
B.
IBC-3
% viable cells
ZY-444 (µM)
C.
IBC-3
SUM149
MB-468
n.s
% viable cells
ZY-444
DMSO
ZY-444
SUM149
IBC-3
MB-468
D.
IBC-3
OCR/ECAR
n.s.
Basal
Maximal
ZY-444
E.
SUM149
MB-468
Oligomycin
FCCP
AA/Rotenone
DMSO
ZY-444
ECAR (mpH/min/ug protein)
ECAR (mpH/min) / ug protein
Time (minutes)

Figure S3

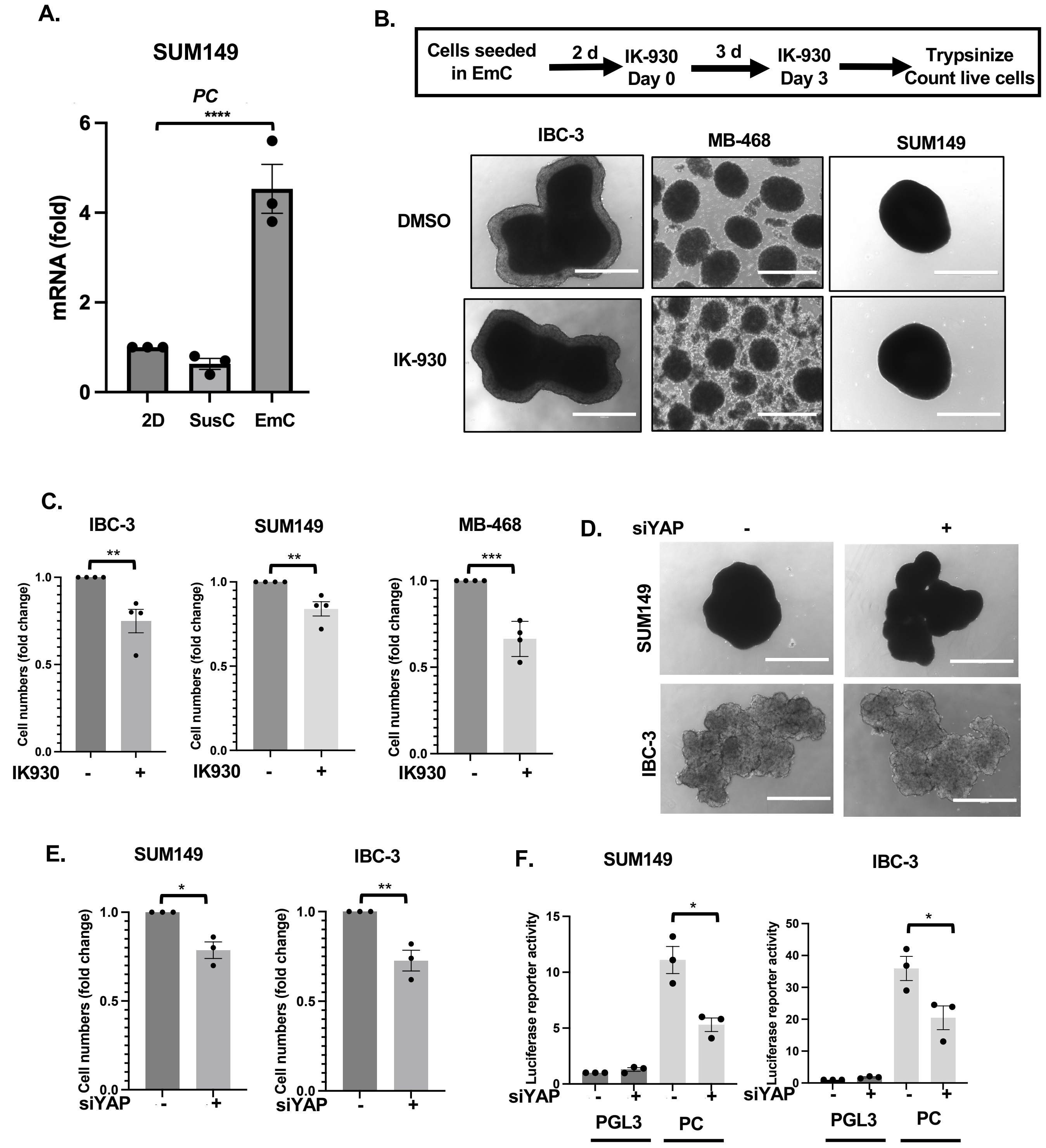

A.
SUM149
PC
****
mRNA (fold)
6
4
2
0
2D
SusC
EmC
B.
Cells seeded in EmC
2 d
IK-930 Day 0
3 d
IK-930 Day 3
Trypsinize Count live cells
IBC-3
MB-468
SUM149
DMSO
IK-930
C.
IBC-3
SUM149
MB-468
**
**
***
Cell numbers (fold change)
1.0
0.5
0.0
IK930
-
+
D.
siYAP
-
+
SUM149
IBC-3
E.
SUM149
IBC-3
*
**
Cell numbers (fold change)
1.0
0.5
0.0
siYAP
-
+
F.
SUM149
IBC-3
Luciferase reporter activity
15
10
5
0
50
40
30
20
10
*
siYAP
PGL3
PC

Figure S4

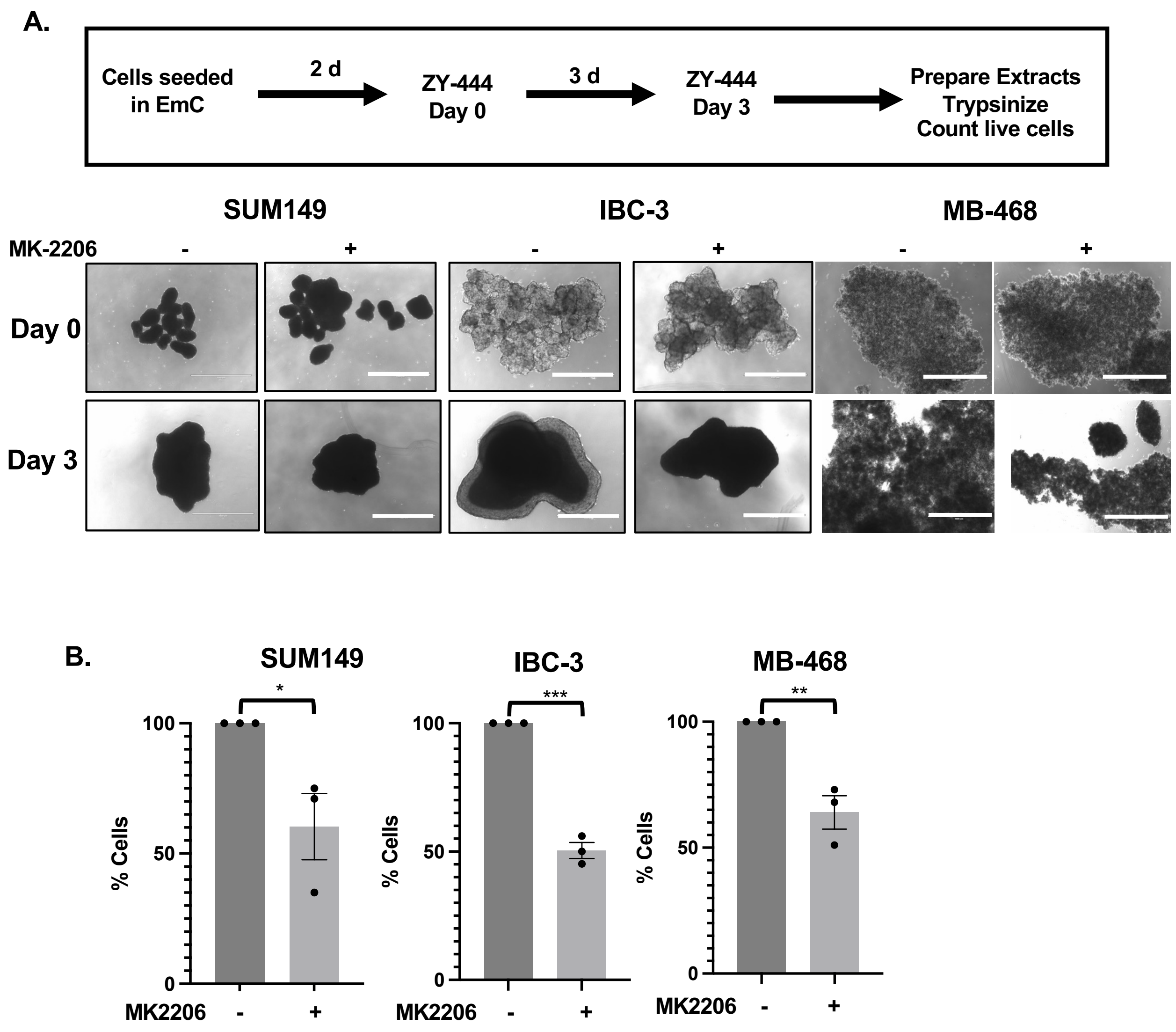
A.
Cells seeded in EmC
2 d
ZY-444 Day 0
3 d
ZY-444 Day 3
Prepare Extracts
Trypsinize
Count live cells
SUM149
IBC-3
MB-468
MK-2206
-
+
Day 0
Day 3
B.
SUM149
IBC-3
MB-468
% Cells
100
50
0
MK2206
*
***
**
